# Green synthesis of gold nanoparticles using aspartame and their catalytic activity for *p*-nitrophenol reduction

**DOI:** 10.1186/s11671-015-0910-7

**Published:** 2015-05-08

**Authors:** Shufen Wu, Songjing Yan, Wei Qi, Renliang Huang, Jing Cui, Rongxin Su, Zhimin He

**Affiliations:** State Key Laboratory of Chemical Engineering, School of Chemical Engineering and Technology, Tianjin University, Tianjin, 300072 People’s Republic of China; Collaborative Innovation Center of Chemical Science and Engineering (Tianjin), Tianjin, 300072 People’s Republic of China; Tianjin Key Laboratory of Membrane Science and Desalination Technology, Tianjin, 300072 People’s Republic of China; School of Environmental Science and Engineering, Tianjin University, Tianjin, 300072 People’s Republic of China; Department of Bioengineering, College of Medicine, Southeast University, Nanjing, 210009 People’s Republic of China

**Keywords:** Gold nanoparticles, Green synthesis, Aspartame, Catalysis

## Abstract

We demonstrated a facile and environmental-friendly approach to form gold nanoparticles through the reduction of HAuCl_4_ by aspartame. The single-crystalline structure was illustrated by transmission electron microscopy (TEM) and X-ray diffraction (XRD). The energy-dispersive X-ray spectroscopy (EDS) and Fourier transform infrared (FTIR) results indicated that aspartame played a pivotal role in the reduction and stabilization of the gold crystals. The crystals were stabilized through the successive hydrogen-bonding network constructed between the water and aspartame molecules. Additionally, gold nanoparticles synthesized through aspartame were shown to have good catalytic activity for the reduction of *p*-nitrophenol to *p*-aminophenol in the presence of NaBH_4_.

**Electronic supplementary material:**

The online version of this article (doi:10.1186/s11671-015-0910-7) contains supplementary material, which is available to authorized users.

## Background

Over the past decade, metallic nanoparticles with large surface-to-volume ratios have attracted steadily growing scientific interest because of their utterly novel characteristics and intriguing applications that are complementary or superior to those of bulk materials [1]. Historically, traditional chemical and physical methods have successfully created well-defined nanoparticles. However, there are drawbacks of these processes including their need of substantial energy and capital, contamination from precursor chemicals, use of toxic solvents, and the generation of hazardous by-products [2].

Hence, during the last two decades, there has been an increasing emphasis on developing straightforward, economically viable, and green synthesis methods for metallic nanoparticles. From an economic and green chemistry perspective, nontoxic solvents, environmentally benign reducing agents, and renewable materials are desirable assets during nanoparticle preparation [3]. In terms of green synthesis methods, water is commonly utilized as an environmentally benign solvent, rather than toxic organic solvents. The most important issue in a green nanoparticle preparation method is the choice of reducing agents and capping materials. The majority of methods in the literature adopt reducing agents such as sodium borohydride (NaBH_4_), hydrazine, and dimethyl formamide (DMF). Notably, all of these chemicals pose potential environmental and biological risks. Moreover, based on the required size ranges and morphologies of the particles, sufficient and special capping agents, which are used to passivate the nanoparticle surface, are indispensable. Thus, there is an apparent inconsistency with the ‘straightforward, economically viable, and green’ principles mentioned above. Considerable efforts have been devoted to investigating green systems. In this regard, biological entities were shown to act as both reducing and stabilizing agents for the green synthesis of metallic nanoparticles. Recently, a vast array of biological resources including plants [4] and plant extracts [5-7], microorganisms [8], fungus [9], glucose [10], chitosan [11], and konica glucomannan (KGM) [12] have been used to synthesize nanocrystals.

Furthermore, inspired by the fact that crystal morphology is always regulated in biology by biomolecules [13], reports to date have focused on using such molecules as templates to synthesize materials with novel nanostructures, for example, simple amino acids [14,15] or polypeptides/proteins [16]. The use of these benign alternatives is ideal for biomedical applications because of their assured eco-friendly property [17]. Unfortunately, the majority of peptides and/or proteins utilized to synthesize metal nanoparticles would be rationally designed and purified before applications. Clearly, this is contrary to the low-cost principium of ‘green’ synthesis.

Herein, a facile approach to rapidly synthesize Au nanoparticles by employing aspartame (APM), a commercially available dipeptide derivative, was developed. Significantly, APM, which is considered to be a biologically compatible chemical, could act as both the reducing and capping agents to generate highly stable Au nanoparticles (APM-AuNPs). Thus, this approach fully complies with the fundamental principles of green chemistry and offers potentially good biocompatibility for biomedical applications. The APM-AuNPs were characterized by ultraviolet-visible (UV-vis) spectroscopy, Fourier transform infrared (FTIR) spectroscopy, dynamic light scattering (DLS), X-ray diffraction (XRD), and transmission electron microscopy (TEM). Finally, the APM-AuNPs were effective in catalyzing the reduction of *p*-nitrophenol (*p*-NP) to *p*-aminophenol (*p*-AP) in the presence of excess NaBH_4_ following pseudo-first-order kinetics. Accordingly, the exploitation of such nanoparticles synthesized by biomolecules could exhibit tremendous opportunities and advantages for biosensing, imaging, or delivery applications.

## Methods

### Materials

Hydrochloroauric acid (HAuCl_4_) and NaBH_4_ were purchased from Aladdin Reagent Company (Shanghai, China). Aspartame (99%) was supplied by Tianfeng Chemical Technology Co., Ltd (China). *p*-Nitrophenol (99%), sodium hydroxide, and other chemicals were of analytical grade. Deionized water was used for all experiments.

### Synthesis of APM-AuNPs

To obtain APM-AuNPs, 0.4 ml of APM aqueous solution (5 mg/ml) and 200 μl of NaOH (100 mM) were added to 9.3 ml of deionized water and incubated in a water bath (Julabo, Seelbach/Black Forest, Germany) (60°C, 200 rpm) for 10 min. Then, 100 μl of HAuCl_4_ (100 mM) was added followed by vigorous stirring for 30 s. Finally, the mixture was incubated for an additional 50 min (60°C, 200 rpm). The effects of several parameters, such as APM concentration (3.4 × 10^−6^ M to 1.7 × 10^−4^ M), HAuCl_4_ concentration (1.0 × 10^−4^ M to 9.0 × 10^−4^ M), pH (2.0 to 12.0), and temperature (25°C/37°C/60°C/90°C) were systematically investigated.

### Characterization of APM-AuNPs

TEM, high-resolution TEM (HRTEM), and energy dispersive X-ray spectroscopy (EDS) characterizations were performed on a JEM-2100 F field emission electron microscope (200 kV). XRD patterns were measured on a Rigaku D/MAX-2500 (Rigaku Co.,Tokyo, Japan) diffractometer with Cu Kα radiation. The particle size range along with the polydispersity of the nanoparticles was determined by DLS using a Malvern Zetasizer Nano system (Malvern Instruments Ltd, Worcestershire, UK). FTIR spectra were recorded in transmittance mode, on a Nicolet Nexus 470 FTIR instrument (Nicolet Co., Madison, WI, USA) using KBr plates. UV-vis absorbance spectra were monitored by a UV-vis spectrophotometer (TU-1810, Purkinje General Co., Ltd, Beijing, China).

### Catalytic activity of APM-AuNPs

For a typical catalysis reaction, 3.0 ml of 4.0 × 10^−3^ M *p*-NP solution was mixed with a freshly prepared NaBH_4_ (1.5 ml, 1.0 × 10^−1^ M), and a volume of APM-AuNPs (1.0 × 10^−3^ M) was added and mixed well to start the reaction.

## Results and discussion

### Characterization of APM-AuNPs

**Figure 1:**
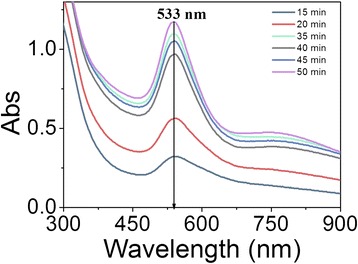
Time evolution of UV-vis spectra during the formation of APM-AuNPs. Conditions: (HAuCl_4_) = 1.0 × 10^−3^ M, (APM) = 6.8 × 10^−6^ M, (NaOH) = 2 mM, 60°C, 200 rpm. Abs, absorption.

The synthetic process of APM-AuNPs was traced by the UV-vis absorption spectra, as shown in Figure 1. With the increase of the reaction time, the intensity of the absorption peak increased gradually and reached a maximum after 50 min. All spectra exhibit an absorption peak around 533 nm without significant peak shifts, which is attributed to the surface plasma resonance (SPR) band of the AuNPs.

**Figure 2:**
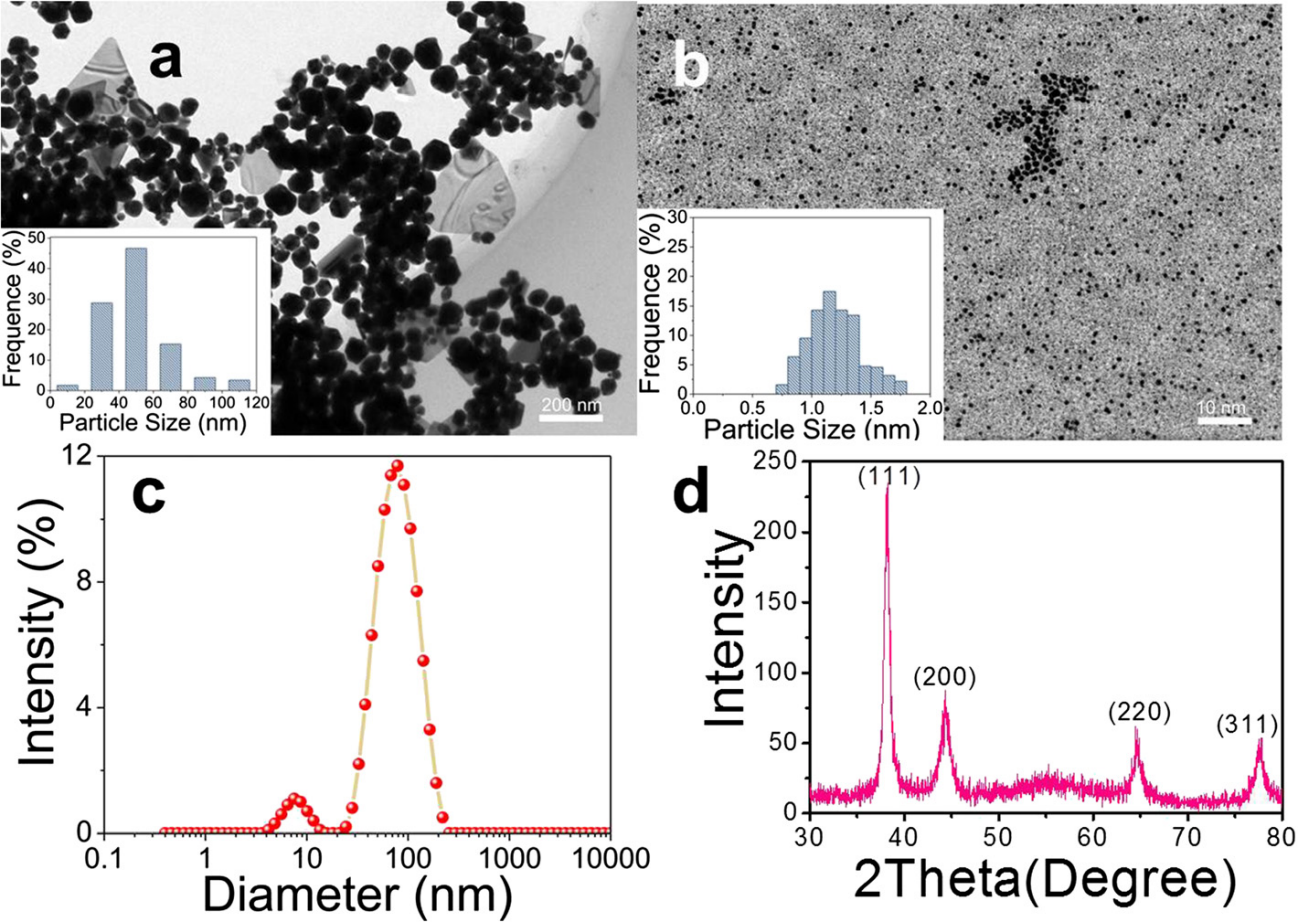
TEM images, DLS, and XRD spectra of APM-AuNPs. TEM images of APM-AuNPs at magnifications **(a)** 200 nm and **(b)** 10 nm with the corresponding particle size distribution histograms; **(c)** Dynamic light scattering spectrum for colloid APM-AuNPs; **(d)** Representative XRD diffraction pattern of APM-AuNPs.

Typical TEM images are displayed in Figure 2a,b. The characteristically spherical nanoparticles with distinct size distributions were observed. It is noteworthy that a few Au nanoplates also appeared; similar morphologies for biosynthetic Au nanoparticles were observed previously [6,7]. DLS is the most versatile and useful technique for measuring the diffusion coefficient of particles in liquids, from which a hydrodynamic diameter can be calculated; in addition, the size distributions and averages can be provided [18]. As shown in Figure 2c, two populations were found, which are supported by the corresponding histograms of the Au nanoparticle size distribution (50 nm and 1.2 nm) (inset in Figure 2a,b). We note that the Au nanoplates were excluded from these statistics. In conclusion, the APM-AuNPs had irregular shapes and a broad size distribution, which is consistent with previous results involving biosynthetic Au nanoparticles [6,7]. The polydispersity in shape and size may be attributed to twinning, a very common process for face-centered cubic (*fcc*) metals.

**Figure 3:**
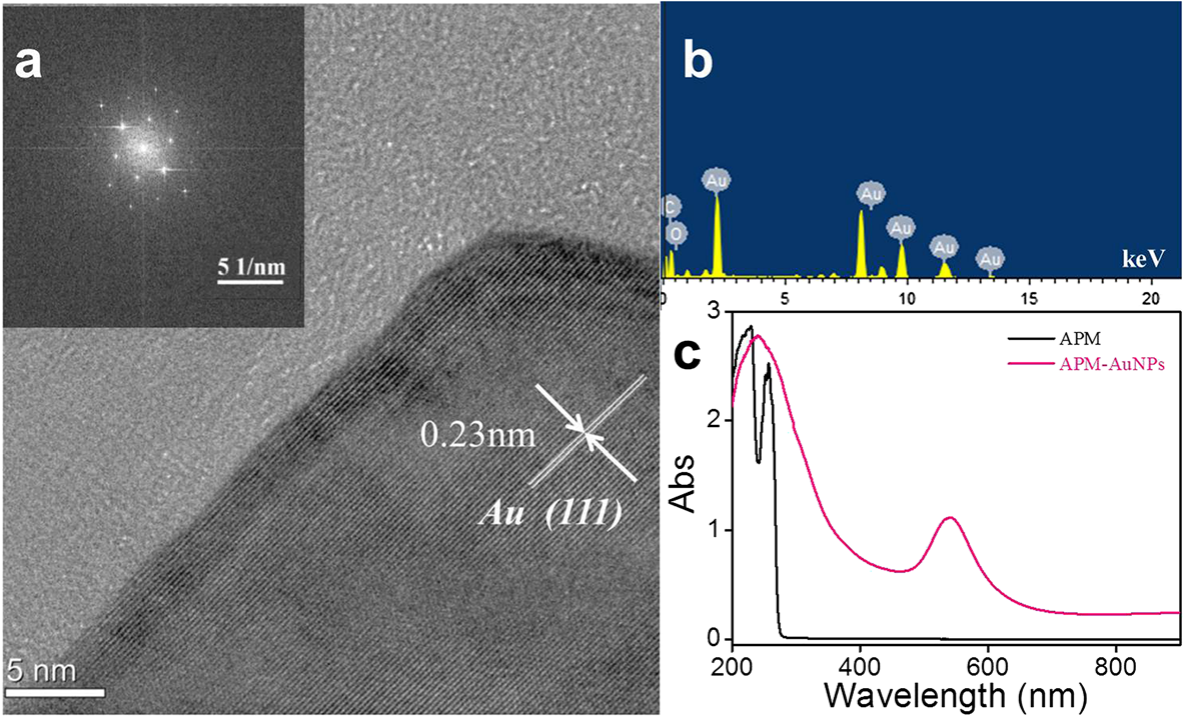
The HRTEM image, TEM-EDS pattern, and UV-visible spectra of APM-AuNPs or APM. **(a)** HRTEM image of APM-AuNPs; **(b)** TEM-EDS pattern of APM-AuNPs; **(c)** UV-visible spectra of APM (black line) and APM-AuNPs (pink line). Abs, absorption.

The crystalline nature of APM-AuNPs was confirmed by powder XRD analysis, as shown in Figure 2d. Clearly, four diffraction peaks can be observed, which could be indexed to the (111), (200), (220), and (311) planes of *fcc* structured metallic gold [10]. We note that the peak corresponding to the (111) plane is more intense than the other planes, suggesting that (111) is the predominant orientation, as supported by the HRTEM (Figure 3a). In addition, the lattice fringes in Figure 3a could be visible with an interplanar distance of 0.23 nm (in agreement with the lattice spacing of the (111) plane), further corroborating the crystalline nature of the formed Au nanoparticles. As shown in Additional file 1: Figure S1, it is clear that the AuNPs are coated with a layer of APM, which suggests that APM can indeed stabilize AuNPs. Furthermore, an analysis of the transmission electron microscopy-energy-dispersive X-ray spectroscopy (TEM-EDS) analysis results (Figure 3b) indicates the presence of strong gold signals, which confirms the reduction of gold ions to elemental gold, and absorption peaks of carbon and oxygen, which confirms that the Au nanoparticles are composed of Au and APM. Herein, a composite consisting of AuNPs and APM was formed in aqueous solution. Additionally, UV-vis spectra of APM and APM-AuNPs solutions (Figure 3c) verified the existence of an interaction between APM and Au nanoparticles. These results confirm that Au nanoparticles were synthesized by APM.

**Figure 4:**
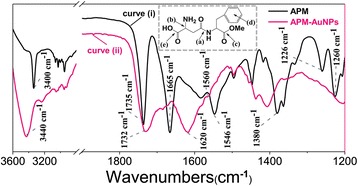
FTIR spectra of APM (black line) and APM-AuNPs (pink line). Inset, chemical structure of APM.

**Figure 5:**
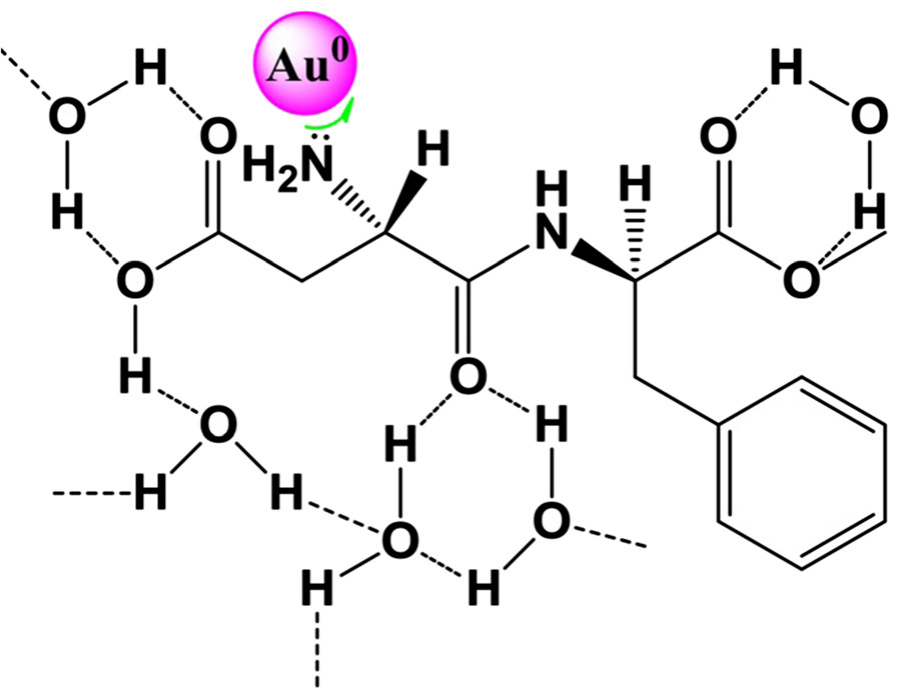
Scheme of cross-linking of APM-AuNPs colloids. The colloids cross via the hydrogen-bonding network constructed by water and APM molecules.

FTIR analysis was used to identify the molecules responsible for the reduction of Au^+^ ions and capping the nanoparticles synthesized by APM. By comparing the FTIR spectra of pure APM and dried APM-AuNPs (Figure 4), the interaction between Au nanoparticles and APM could be confirmed. As noted (inset, Figure 4, upper), the functional groups leading to characteristic IR spectra of APM included the (a) amide group, (b) primary amino group, (c) ester moiety, (d) monosubstituted benzene ring, and (e) carboxylic group. The sharp peaks at 1,665 cm^−1^ (curve (i)) and 1,546 cm^−1^ (curve (i)) were assigned to the C = O stretching vibration (amide I) and a mixed vibration of NH deformation and CN stretch (amide II) in amides [19,20], respectively. In addition, the IR bands at approximately 3,400 cm^−1^ (3,400 cm^−1^ in curve (i) and 3,440 cm^−1^ in curve (ii)) could also be observed [20-22], which may be assigned to an O-H stretching vibration in the carboxyl moiety, NH stretching of NH_2,_ or CH stretching. By contrast, the absorption band corresponding to amide II on the IR spectrum of dried APM-AuNPs (curve (ii)) was broadened and blueshifted to 1,560 cm^−1^, whereas the band corresponding to amide I was also broadened but redshifted to 1,620 cm^−1^. Generally, such broadening and shifting of the vibrational spectra have been ascribed to the formation of hydrogen-bonded molecular complexes [23]. Another feature of the gold particles was the presence of a strong and broad band centered at 3,440 cm^−1^. On the basis of literature values [24], this band was attributed to be the various O-H stretching vibrations. The literature further corroborated the existence of possible O-H----O hydrogen-bonding interactions, as suggested in Figure 5, which might be associated with the ester-moiety stretching [20]. Specifically, the peaks at 1,380 cm^−1^, 1,226 cm^−1^, and 1,260 cm^−1^ were assigned to the bending of methyl group, C-O stretching of ester, and the methoxyl group [19], respectively. Likewise, another medium IR band cluster ranging from 2,800 to 3,200 cm^−1^ (curve (i) and (ii), marked in Figure 4, left) and attributed to the aromatic CH of the monosubstituted benzene [25] is displayed. It should be noted that the intense bands at 1,735 cm^−1^ (curve (i)) and 1,732 cm^−1^ (curve (ii)), which are identified as the C = O stretch in the ester moiety, suggest that no demethylation occurred via a classical intermolecular hydrolysis. This was verified via the result of the HPLC profiles (Additional file 1: Figure S2), which indicated that no degradation of APM occurred during 2 days at acidic (pH 2.0) or very basic pH conditions (pH 13.0) [26]. It was thus concluded that APM could serve as a reducing agent.

### Influence of experimental parameters

It is well known that the optical properties of metal nanoparticles depend strongly on their size and shape and the surrounding medium [27]. To obtain the best Au nanoparticles, the effects of the concentration of reactants, pH, and temperature on the formation of APM-AuNPs have been investigated through UV-vis measurements.

As can be observed (Additional file 1: Figure S3), at a constant concentration of HAuCl_4_, the solutions containing APM (5 mg/ml, 0.2/0.4/1.0 ml, marked with I, II, and III, respectively) showed firebrick, red wine, and blue coloring, which signified that APM-AuNPs were formed with different sizes and shapes. This was further confirmed by the UV-vis absorption spectra (Additional file 1: Figure S4a), compared with the UV-vis band of II (peaked at 533 nm), those of I and III were clearly broadened and highly redshifted (572 nm and 548 nm, respectively), suggesting a possible increase in the shape diversity and particle size because of extrinsic size effects [28]. Similarly, strong and broad bands occurred with increasing APM concentration (Additional file 1: Figure S4b, 2 to 10 ml). Likewise, at a constant aqueous concentration of APM, higher concentrations of HAuCl_4_ gave much broader peaks with higher intensities (Additional file 1: Figure S4c), indicating a possible increase in the particle size [28]. Apparently, a rational proportion of APM and HAuCl_4_ are critical to control the size and shape of AuNPs. In general, a low concentration of APM was not sufficient to complete the bio-reduction, whereas excess reducing agent could result in either an additional interaction among the surface capping molecules or a secondary reduction process on the surface of nuclei, which gave rise to larger particles and irregular shapes (e.g., triangular or hexagonal).

The influence of pH was also investigated and the results are shown in Figure S4d (see Additional file 1). On the basis of the UV-vis spectra, the addition of 200 μl of NaOH (100 mM) to the ‘original’ reaction system (pH 7.10) resulted in an ideal SPR band (absorption peak located at 533 nm).

Furthermore, the SPR spectral profiles of APM-AuNPs synthesized at different temperatures (25°C, 37°C, 60°C, and 90°C) are shown in Figure S5 (see Additional file 1). The relatively ideal SPR peak occurred at approximately 533 nm on account of the blueshift effect with the decrease in particle size [28]. As expected, the time required to complete the reaction increased gradually as the temperature was lowered. It was found that the reactions finished within 1 h at 60°C or 90°C, whereas 1 day or more time was needed at 37°C or 25°C. Au nanoparticles synthesized at 60°C demonstrated good stability, as indicated by the absence of a solution color and the inability to detect shifts of the absorption peaks after 4 months. However, APM-AuNPs under 90°C aggregated within several hours. Therefore, 60°C was chosen as the optimal reaction temperature.

### Schematic model of APM-AuNPs synthesis

Based on the aforementioned results, a possible mechanism for forming APM-AuNPs is proposed in Figure 5. Although the SH moiety is known to play a critical role in the reduction of Au^3+^ species, the carboxylic acid (COOH) group could spontaneously reduce aqueous chloroaurate ions leading to the formation of Au nanoparticles [29]. Additionally, it is well known that amine groups can bind to gold nanoparticles [30]. Thus, it could be deduced that the reduction of Au^3+^ by APM occurred via the COOH group, and the primary NH_2_ bound to the surface of Au nanoparticles. Presumably, the Au nanoparticles become stabilized through the hydrogen-bonding network constructed by water and APM molecules, as shown in Figure 5. To verify this assumption, sufficient quantities of DMSO were added to the APM-AuNPs solution with the aim of disrupting the hydrogen bonds, since DMSO is recognized as a strongly polar solvent [31]. As expected, black Au precipitates appeared following the addition of DMSO, as shown in Figure S6a (marked with I, Additional file 1). To further investigate this assumption, the pH of APM-AuNPs solution was raised to 12.0, which is higher than the p*K*a values of APM (3.1 and 7.9) [32]. Notably, the aqueous solution turned light blue (see Figure S6b, marked with I, Additional file 1), indicating that the aggregation occurred [28]. Theoretically, most of the COOH groups of APM are dissociated into in very basic condition. The presence of the COO^−^ groups would result in the disassembling of the hydrogen-bonding network because of Coulomb repulsion [29]. Therefore, the hydrogen-bonding network played a crucial role in the stabilization of the APM-AuNPs.

### Application of APM-AuNPs for the catalytic reduction of *p*-NP

**Figure 6:**
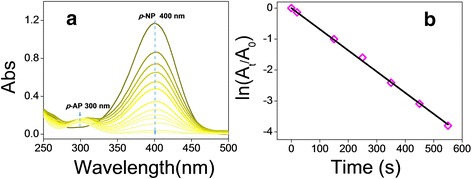
Absorption spectra and plots of ln(A_0_/A_t_) against time. **(a)** Green line shows the UV-vis spectra of *p*-nitrophenolate following the addition of NaBH_4_ solution but without the addition of APM-AuNPs, and others showed the time-dependent UV-vis spectra of *p*-NP reduction by APM-AuNPs with excess NaBH_4_ solution. **(b)** Plot of ln(A_0_/A_t_) against time for APM-AuNPs catalytic reduction of *p*-NP, with conditions: (*p*-NP) = 6.3 × 10^−4^ M, (NaBH_4_) = 7.5 × 10^−3^ M, (APM-AuNPs) = 2.3 × 10^−4^ M and *T* = 25°C. Abs, absorption.

Metallic nanoparticles have been the subject of intense research during recent years, because of their potential applications to activate or catalyze reactions that are otherwise unfeasible. In particular, redox reactions catalyzed by nanoparticles have been extensively investigated. The reduction of *p*-NP to *p*-AP with an excess amount of NaBH_4_, which could be easily monitored by UV-vis spectroscopy, has been identified as a model reaction. To this end, we selected this system to quantitatively evaluate the catalytic function of the APM-AuNPs. The *p*-NP solution has an absorption peak at 317 nm (Additional file 1: Figure S9a). Upon the addition of a fresh NaBH_4_ solution, an absorption peak at a 400 nm (indicating the appearance of *p*-nitrophenolate ion) was immediately seen, which was concomitant with a color change from light yellow to yellow-green. Without the APM-AuNPs, the absorption peak at 400 nm remained unaltered for a long duration, suggesting that no reaction occurred in the absence of catalysis [33]. However, after the APM-AuNPs solution was introduced, the absorption peak at 400 nm decreased gradually and a new peak at 300 nm (Figure 6a) appeared, which was followed by a fading and eventual bleaching of the yellow-green color of *p*-nitrophenolate ion. For a typical measurement, the reaction progress was monitored by the time-dependent UV-vis absorption spectra (Figure 6a). When the concentration of NaBH_4_ is much larger than that of *p*-NP, a pseudo-first-order kinetics was used to evaluate the rate constant [34]. In Figure 6b, a good linear correlation between ln(A_t_/A_0_) and reaction time were observed, and the corresponding rate constant was calculated to be 6.84 × 10^−3^ s^−1^, which is comparable to that of other AuNPs for the reduction of *p*-NP in the presence of NaBH_4_ [35,36].

The catalytic property of APM-AuNPs was further investigated. The reaction above was performed at temperatures of 37°C, 45°C, and 60°C with variable concentrations of APM-AuNPs. As shown in Additional file 1: Figure S7, the total reaction time was inversely proportional to the temperature and the concentrations of APM-AuNPs. The corresponding rate constants (*k*) are given in Figure S8b,c,d (see Additional file 1). Clearly, the value of *k* maintained proportional to the temperature and the concentrations of the APM-AuNPs. Moreover, the apparent activation energy (*Ea*) for this reaction was calculated to be 108.83 kJ/mol. Thus, it can be deduced that the reduction of *p*-NP preferentially took place on the most exposed facet (111).

## Conclusions

In summary, a facile and eco-friendly approach for the synthesis Au nanocrystals was explored by utilizing APM both as a reducing agent and a capping agent. The TEM, XRD, and EDS analyses verified the formation of APM-AuNPs, and the spectroscopic techniques (UV-visible and FTIR) and visual observations proved that APM played a pivotal role in the reduction and stabilization of gold crystals. Additionally, the catalytic function of APM-AuNPs to reduce *p*-NP to *p*-NA in the presence of NaBH_4_ was validated. Further investigation will be performed to explore the biomedical and biosensor applications of APM-AuNPs.

## Additional file

Additional file 1:
**Supporting information green synthesis of gold nanoparticles using aspartame and their catalytic activity for p-nitrophenol reduction.** A document showing supplementary figures.
