# Supplementary material for: Green synthesis of gold nanoparticles using aspartame and their catalytic activity for p-nitrophenol reduction
Source: Nanoscale Res Lett. 2015 May 8;10:213. doi: 10.1186/s11671-015-0910-7 (PMC4431991; doi:10.1186/s11671-015-0910-7)
Supplement: Additional file 1: — Supporting information green synthesis of gold nanoparticles using aspartame and their catalytic activity for p-nitrophenol reduction. A document showing supplementary figures. [file 11671_2015_910_MOESM1_ESM.doc]

***Supporting Information for***

**Green synthesis of gold nanoparticles using aspartame and their catalytic activity for *p*-nitrophenol reduction**

Shufen Wu^1^, Songjing Yan^1^, Wei Qi^*1, 2, 3^, Renliang Huang^4^, Jing Cui^5^, Rongxin Su^1,2,3^, Zhimin He^1,2^

* Correspondence: qiwei@tju.edu.cn.

^1^ State Key Laboratory of Chemical Engineering, School of Chemical Engineering and Technology, Tianjin University, Tianjin 300072, People’s Republic of China.

^2^ Collaborative Innovation Center of Chemical Science and Engineering (Tianjin), Tianjin 300072, People’s Republic of China.

^3^ Tianjin Key Laboratory of Membrane Science and Desalination Technology, Tianjin, 300072, People’s Republic of China.


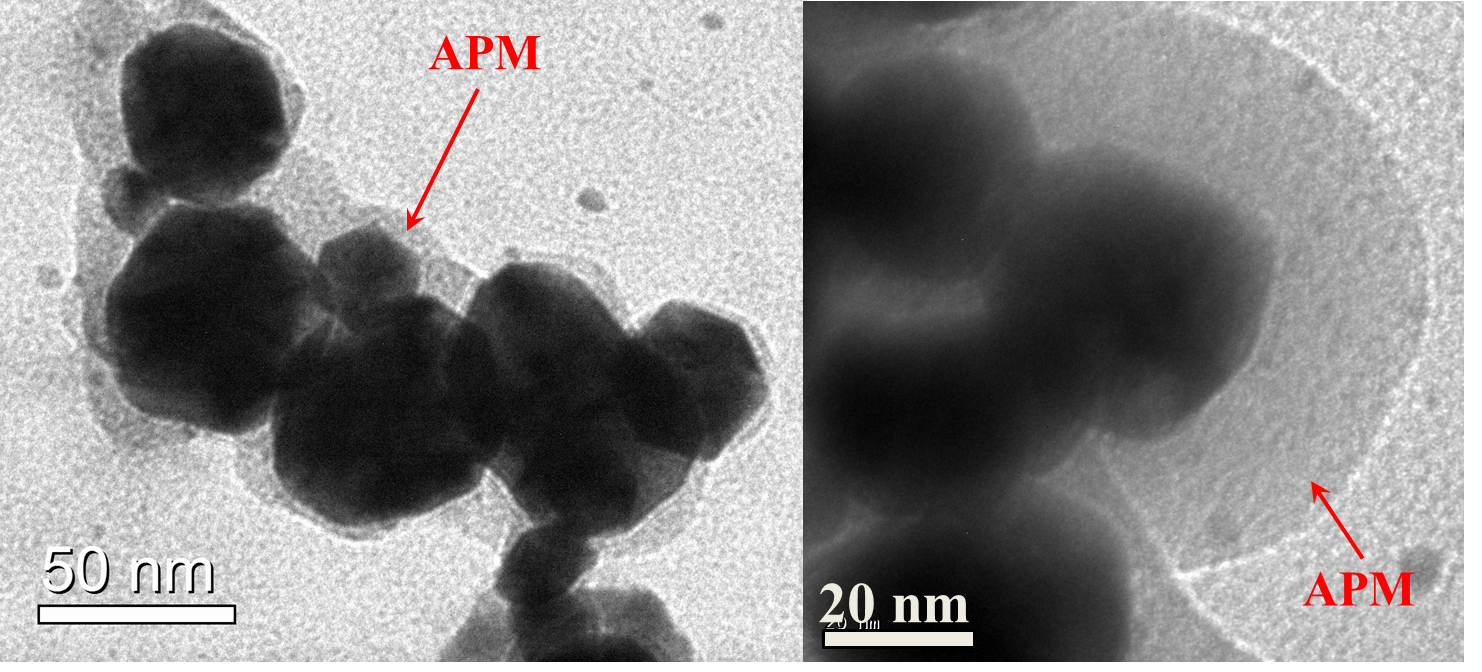


**Figure S1.** TEM images of APM-AuNPs.


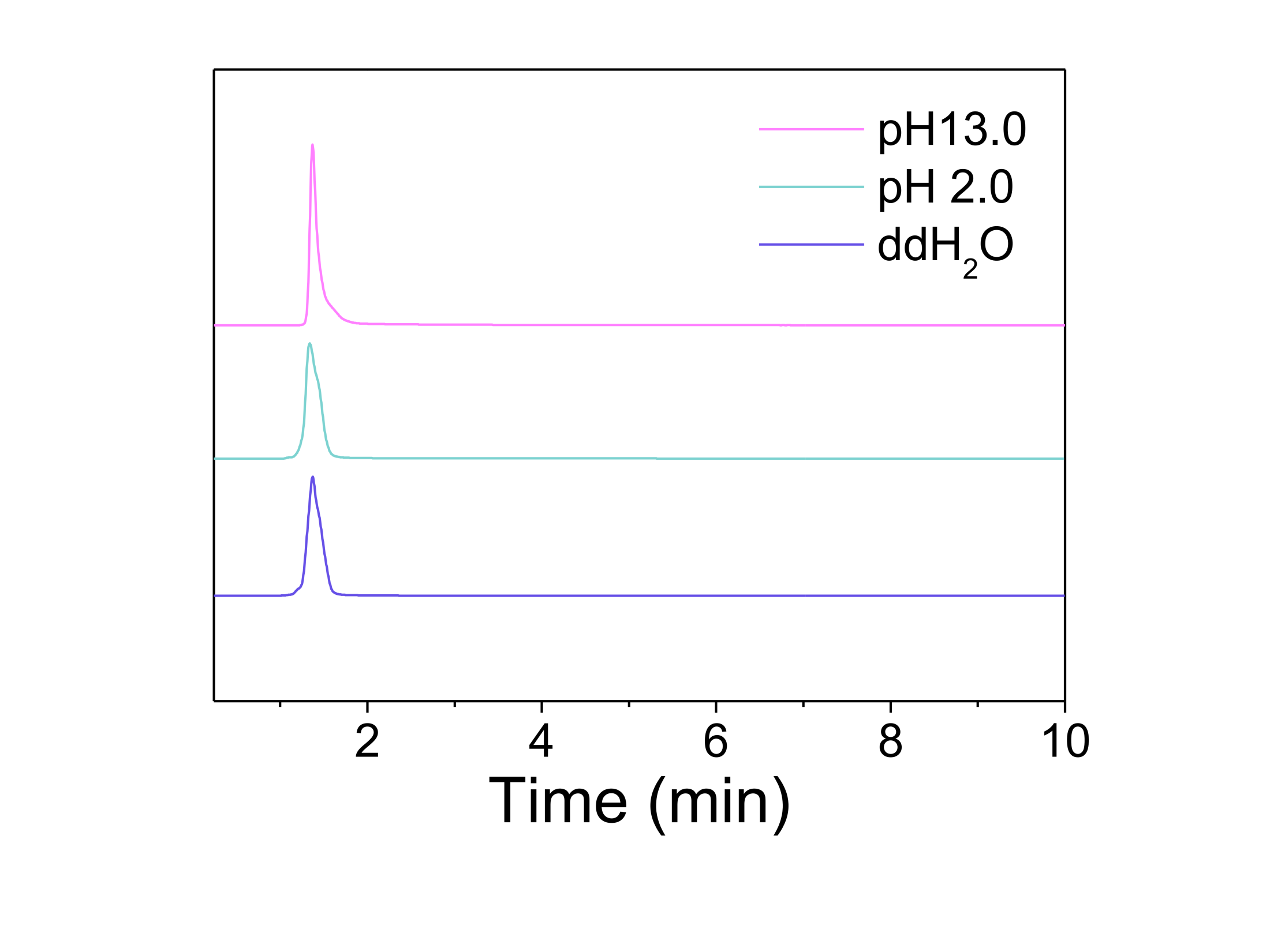


**Figure S2.** HPLC chromatograms of APM at different conditions: pH 13.0, pH 2.0 and dissolved in ddH_2_O.


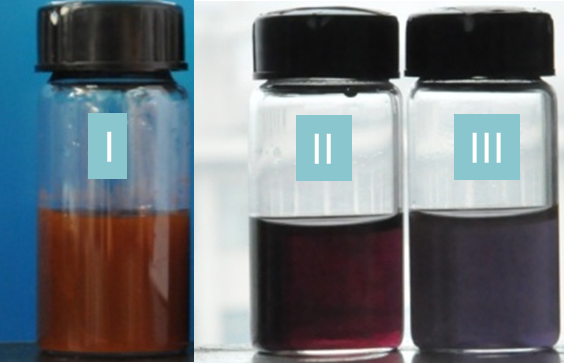


**Figure S3.** The images of colloid APM-AuNPs. Conditions: [HAuCl_4_] = 1.0×10^-3^ M, APM (5 mg/ml) (I) 0.2 ml; (II) 0.4 ml; (III) 1.0 ml.


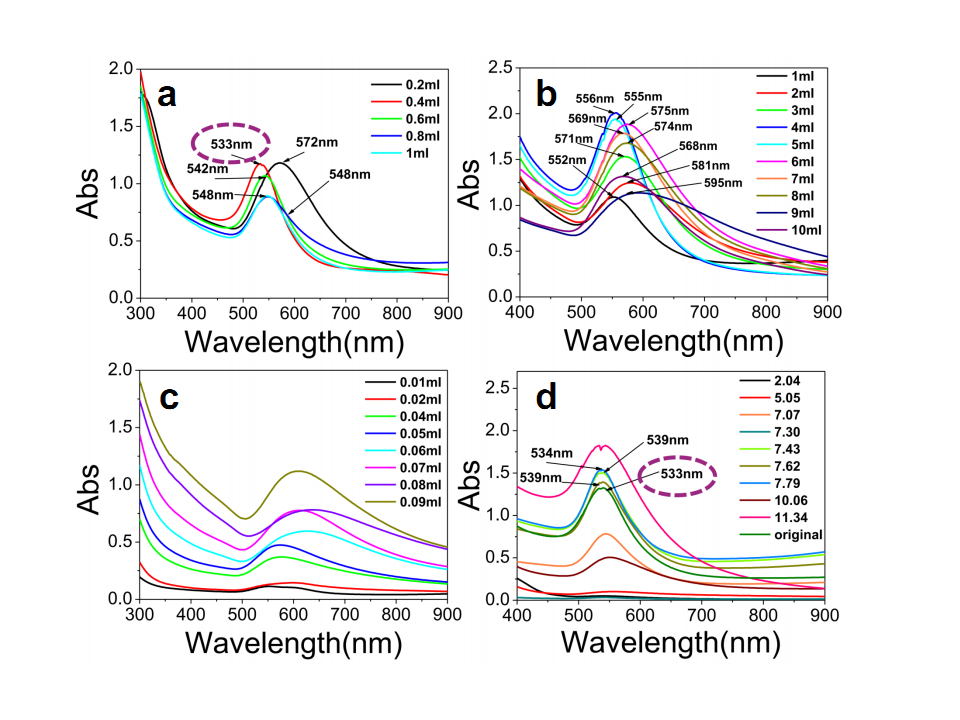


**Figure S4.** (a) and (b) Effect of the APM concentration on the UV-vis spectra of APM-AuNPs, (c) Effect of the HAuCl_4_ concentration on the UV-vis spectra of APM-AuNPs, and (d) Effect of pH on the UV-vis spectra of APM-AuNPs. Conditions: (a) and (b), [HAuCl_4_] = 1.0×10^-3^ M, [APM] = 3.4×10^-6^ M – 1.7×10^-4^ M; (c) [APM] = 6.8×10^-6^ M, [HAuCl_4_] = 1.0×10^-4^ M – 9.0×10^-4^ M; (d) [APM] = 6.8 ×10^-6^ M, [HAuCl_4_] = 1.0×10^-3^ M, the “original” indicates the sample without any adjustment of the pH (the real pH value was 7.10).


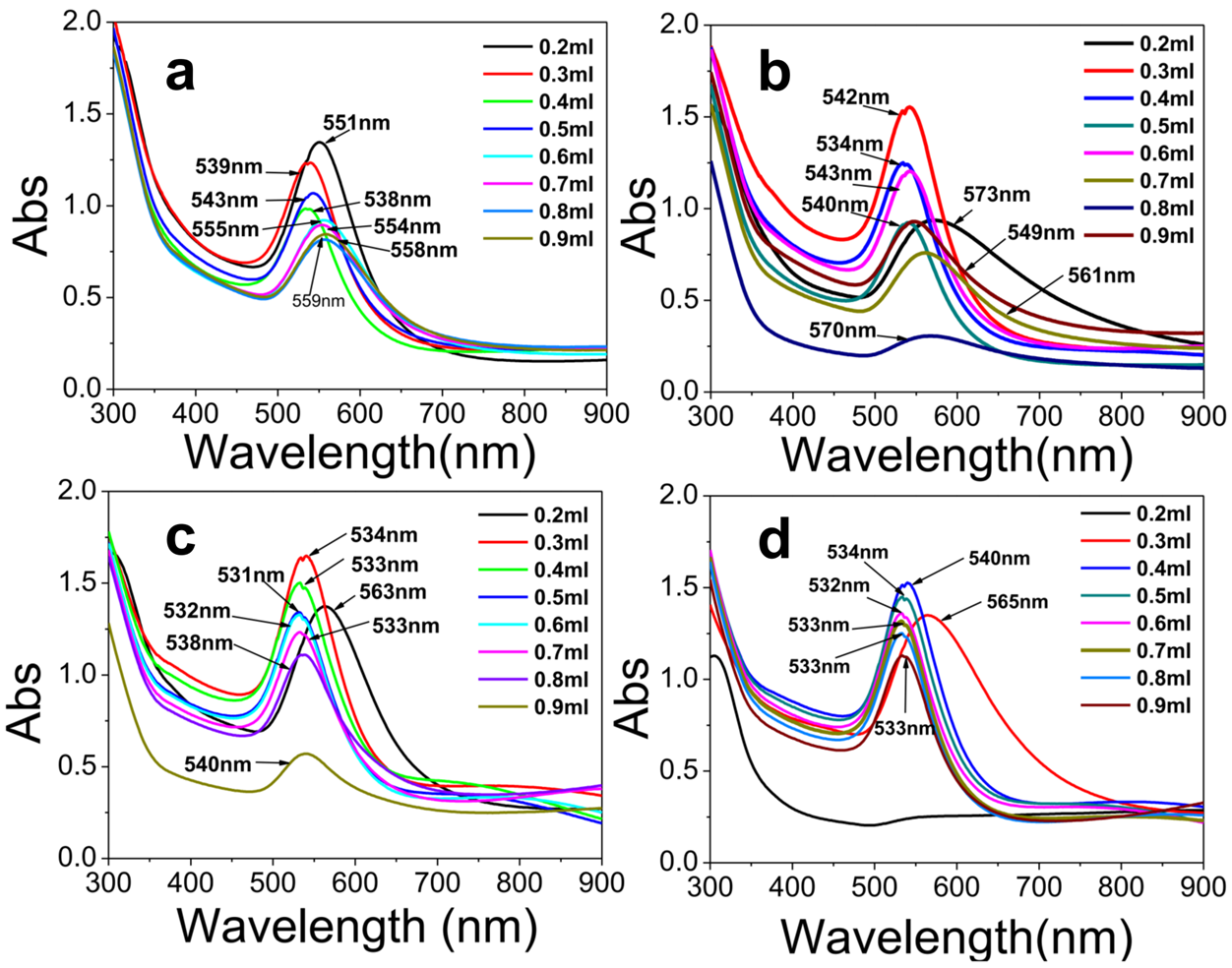


**Figure S5.** UV-vis spectra of APM-AuNPs prepared under different reaction temperatures (a) 25 ^o^C, (b) 37 ^o^C, (c) 60 ^o^C, (d) 90 ^o^C. Conditions: [HAuCl_4_] = 1.0×10^-3^M, [APM] = 3.4×10^-6^ M – 1.53×10^-5^ M, 200 rpm.


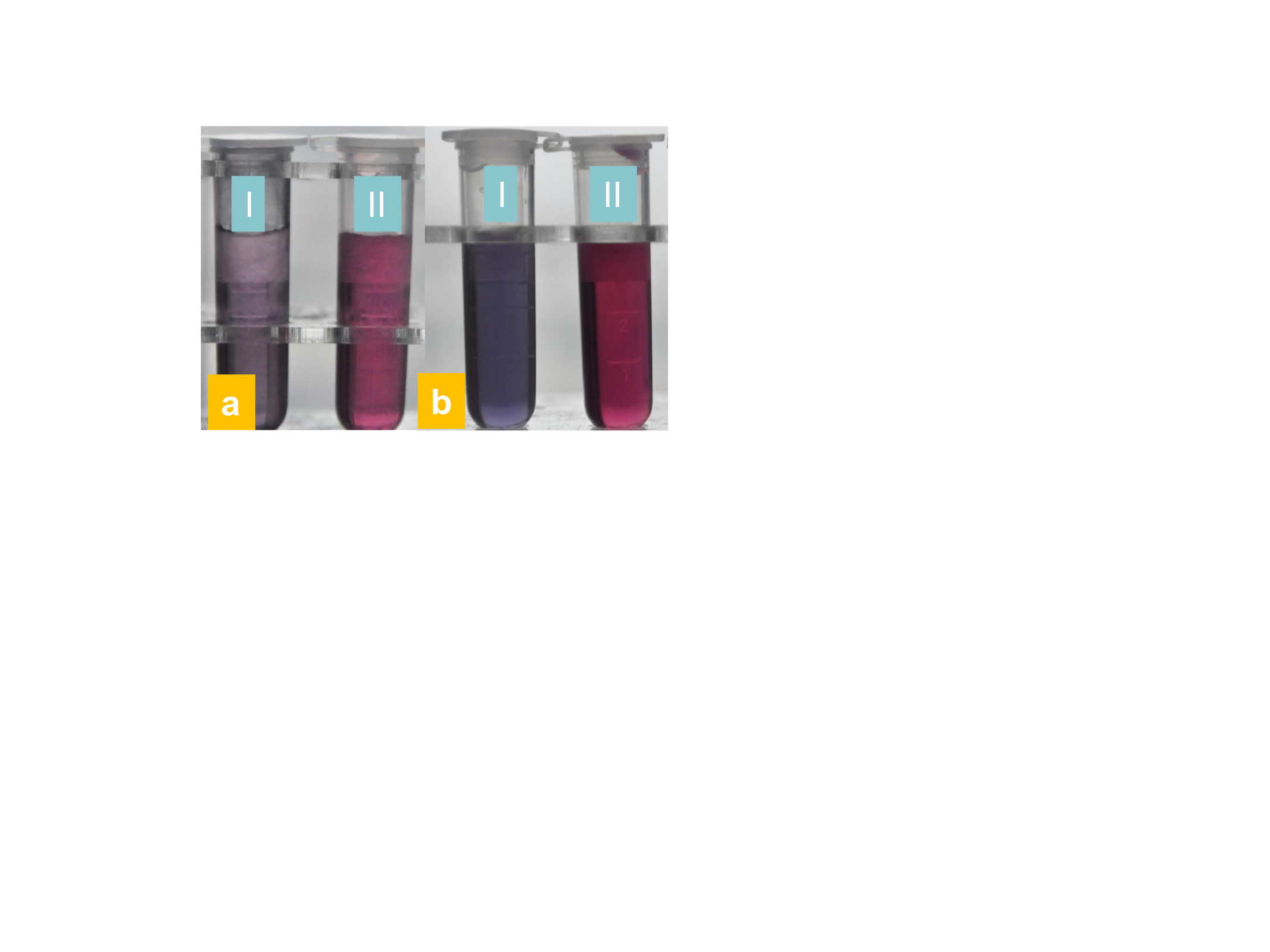


**Figure S6.** Images of colloid APM-AuNPs. (a) The pH value adjusted to 12.0 is marked with I and the sample without any adjustment of the pH is marked with II; (b) 2 ml of DMSO is added to the sample marked with I and the sample without DMSO is marked with II.


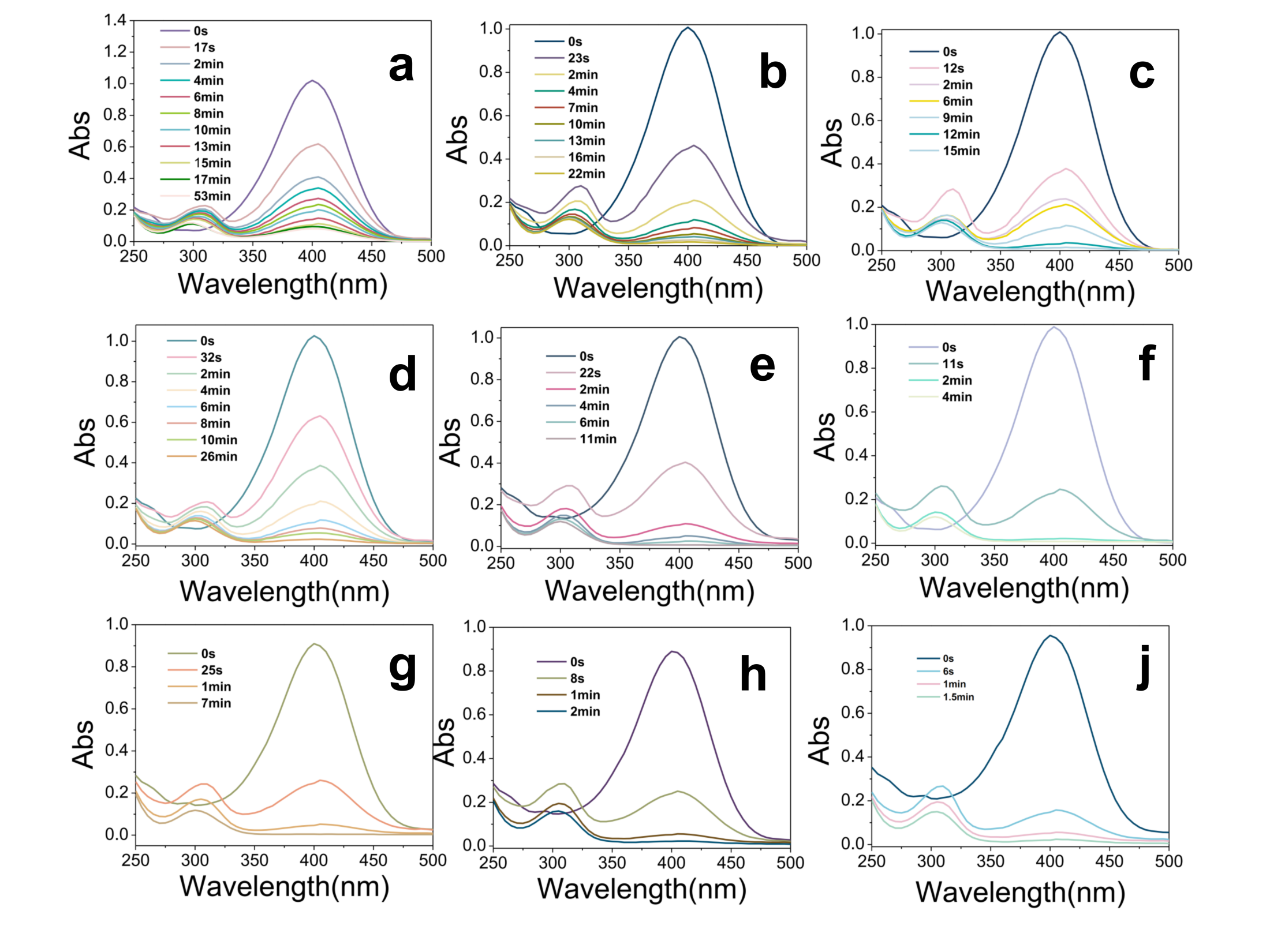


**Figure S7.** Time-dependent UV-vis spectra for the reduction of *p*-NP in the presence of NaBH_4_ performed at (a), (d) and (g) 37 ^o^C; (b), (e) and (h) 45 ^o^C; (c), (f) and (j) 60 ^o^C with different concentrations of APM-AuNPs. [*p*-NP] = 6.3 ×10^-4^M; [NaBH_4_] = 7.5 × 10^-4^ M; [APM-AuNPs]: (a–c) 2.3 ×10^-4^ M; (d–f) 3.8 ×10^-4^ M; (g–j) 5.4 ×10^-4^ M.

**Figure S8.** (a) (
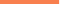
) indicates the UV-vis spectra of *p*-NP without the adding of a NaBH_4_ solution; (
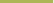
) indicates the UV-vis spectra of *p*-NA following the adding of a NaBH_4_ solution but without the addition of APM-AuNPs. (b) Plot of ln[*p*-NP] against time for the reduction of 4-nitrophenol performed at 37 ^o^C, 45 ^o^C and 60 ^o^C, with conditions: [*p*-NP] = 6.3 ×10^-4^ M, [NaBH_4_] = 7.5 ×10^-4^ M, [APM-AuNPs] = 2.3 ×10^-4^ M. (c) Plot of ln[*p*-NP] against time for the reduction of *p*-NP performed at 37 ^o^C, 45 ^o^C as well as 60 ^o^C, with conditions: [*p*-NP] = 6.3 ×10^-4^ M, [NaBH_4_] = 7.5 ×10^-4^ M, [APM-AuNPs] = 3.8 ×10^-4^ M. (d) Plot of ln[*p*-NP] against time for the reduction of *p*-NP performed at 37 ^o^C, 45 ^o^C as well as 60 ^o^C, with conditions: [*p*-NP] = 6.3×10^-4^ M, [NaBH_4_] = 7.5 ×10^-4^ M, [APM-AuNPs] = 5.4 ×10^-4^ M**.**
